# Lack of association between lipoprotein(a) genetic variants and subsequent cardiovascular events in Chinese Han patients with coronary artery disease after percutaneous coronary intervention

**DOI:** 10.1186/1476-511X-12-127

**Published:** 2013-08-27

**Authors:** Zhi-Gen Li, Guang Li, Ying-Ling Zhou, Zhu-Jun Chen, Jun-Qing Yang, Ying Zhang, Shuo Sun, Shi-Long Zhong

**Affiliations:** 1 Department of Cardiology, Guangdong Cardiovascular Institute, Guangdong Academy of Medical Sciences, Guangdong General Hospital, 96 Dongchuan Road, Guangzhou 510007, China; 2 Medical Research Center, Guangdong Cardiovascular Institute, Guangdong Academy of Medical Sciences, Guangdong General Hospital, 96 Dongchuan Road, Guangzhou 510007, China; 3 Department of Geriatrics, Guangzhou First People’s Hospital, Guangzhou Medical University, 1 Panfu Road, Guangzhou 510045, China

**Keywords:** Coronary artery disease, Lipoprotein(a), Major adverse cardiovascular events, Percutaneous coronary intervention, Single-nucleotide polymorphism

## Abstract

**Background:**

Elevated lipoprotein(a) [Lp(a)] levels predict cardiovascular events incidence in patients with coronary artery disease (CAD). Genetic variants in the rs3798220, rs10455872 and rs6415084 single-nucleotide polymorphisms (SNPs) in the Lp(a) gene (*LPA*) correlate with elevated Lp(a) levels, but whether these SNPs have prognostic value for CAD patients is unknown. The present study evaluated the association of *LPA* SNPs with incidence of subsequent cardiovascular events in CAD patients after percutaneous coronary intervention (PCI).

**Methods:**

TaqMan SNP genotyping assays were performed to detect the rs6415084, rs3798220 and rs10455872 genotypes in 517 Chinese Han patients with CAD after PCI. We later assessed whether there was an association of these SNPs with incidence of major adverse cardiovascular events (MACE: cardiac death, nonfatal myocardial infarction, ischemic stroke and coronary revascularization). Serum lipid profiles were also determined using biochemical methods.

**Results:**

Only the rs6415084 variant allele was associated with higher Lp(a) levels [41.3 (20.8, 74.6) vs. 18.6 (10.3, 40.9) mg/dl, p < 0.001]. During a 2-year follow-up period, 102 patients suffered MACE, and Cox regression analysis demonstrated that elevated Lp(a) (≥30 mg/dl) levels correlated with increased MACE (adjusted HR, 1.69; 95% CI 1.13-2.53), but there was no association between *LPA* genetic variants (rs6415084 and rs3798220) and MACE incidence (p > 0.05).

**Conclusions:**

Our data did not support a relationship between genetic *LPA* variants (rs6415084 and rs3798220) and subsequent cardiovascular events after PCI in Chinese Han CAD patients.

## Introduction

Cardiovascular disease is the leading cause of morbidity and mortality worldwide. In past decades, multiple cardiovascular disease risk factors have been identified and used for risk stratification and outcome evaluations; lipoprotein (a) (Lp(a)) is one of the most attractive and promising risk factors.

Lp(a) is a plasma lipoprotein consisting of a cholesterol-rich low density lipoprotein (LDL) particle with one apolipoprotein B100 (apoB100) molecule and an additional apolipoprotein(a) (apo(a)) protein attached via a disulfide bond [1]. Previous studies demonstrated that elevated Lp(a) levels are associated with increasing incidence and severity of cardiovascular diseases [2-7]. Furthermore, elevated baseline Lp(a) levels predict the subsequent cardiovascular event incidence in patients with coronary artery disease (CAD) after percutaneous coronary intervention (PCI) [8-10].

Serum Lp(a) levels are largely affected by Lp(a) gene (*LPA*) variations, which varies widely across different races [11-13]. Recently, many single-nucleotide polymorphisms (SNPs) have been identified in *LPA*[11,12,14-16]. Among these polymorphisms, rs3798220 and rs10455872 are two common *LPA* variants that have been studied intensively. Rs3798220 and rs10455872 are short variations in the 5’ haplotype block of *LPA,* which is located at the 6q27 chromosomal region. Both of these variations are strongly associated with serum Lp(a) levels in Caucasians, and each rs10455872 (G; 7.0% frequency) and rs3798220 (C; 2.0% frequency) minor allele increased log Lp(a) by 1.18 and 1.27 standard deviation units [17], respectively. A series of case–control studies demonstrated that rs10455872 and rs3798220 are significantly associated with CAD and myocardial infarction risk and are also associated with atherosclerotic burden, obstructed coronary artery number and an earlier CAD diagnosis [17-19]. However, whether these two *LPA* SNPs correlate to subsequent cardiovascular event risk for CAD patients after PCI is unknown. The present study was therefore aimed to explore the association between the two aforementioned SNPs in addition to another *LPA* SNP rs6415084, which is also a short variation in the 5’ haplotype block of *LPA* and correlates with Lp(a) levels [12], and the incidence of subsequent cardiovascular events in Chinese Han patients with CAD after PCI.

## Methods

### Study population

In total, five hundred and seventeen Chinese Han patients from Guangdong General Hospital were sequentially enrolled during May 2009 and August 2010. All of the patients were between 18 and 80 years old, unrelated Han Chinese male and female CAD patients. All of the patients received PCI before discharge. We excluded patients for any of the following: contraindication to aspirin or clopidogrel therapy, pre-existing bleeding disorders, pregnancy, lactating or planning to become pregnant, advanced cancer, hemodialysis and unsuccessful genotyping. This study has been registered in the Chinese Clinical Trial Registry (registration number: ChiCTR-OCH-11001198). All of the participants provided written informed consent for genetic analysis. The Guangdong general hospital ethics committee approved the study.

### Genotyping

Genomic DNA was extracted from 4 ml EDTA-anticoagulated blood with the PureGene DNA isolation kit (Gentra Systems, Minnesota, USA) according to the manufacturer’s recommendations. Rs6415084 rs3798220 and rs10455872 of *LPA* were determined for each patient using the TaqMan genotype discrimination assay (Applied Biosystems, California, USA) following the manufacturer’s instructions.

### Lipid profile determination

Serum lipid profiles including total cholesterol, triglycerides, LDL-cholesterol (LDL-C), high-density lipoprotein cholesterol (HDL-C), apolipoproteinA (apoA), apolipoproteinB (apoB) and Lp(a) were determined by biochemical methods on the second day of each patients’ admission. Lp(a) was measured in serum samples using sandwich enzyme-linked immunosorbent assays (ELISA) [Lp(a) ELISA kit, Yaji Biosystems, Shanghai, China] and a SYNCHRON LX20 UniCel DxC800 analyzer (Beckman Coulter Inc., USA); less than 30 mg/dl was considered within the normal range.

### Data collection clinical follow-up

Baseline risk factors, coronary angiographic findings and medication use were recorded for all of the enrolled patients. Follow-up information was collected based on inpatient and outpatient hospital visits as well as telephone contacts with the patients or their families 6, 12, 18, 24 months after PCI. The clinical end point was the cumulative incidence of major adverse cardiovascular events (MACE) including cardiac death, nonfatal myocardial infarction (MI), ischemic stroke (CT or MR scan confirmed) and coronary revascularization. Coronary revascularization included PCI (including atherectomy, balloon angioplasty and stenting) or coronary artery bypass grafting in the follow-up period. For subjects with multiple events, only the first event was considered for analysis.

### Data analysis

Categorical data are reported as frequencies, and differences between groups were compared with the χ^2^ test. Fisher exact tests were used when the expected cell frequencies were less than 5. Continuous data are reported as the mean (standard deviation) or median (interquartile range, quartile1-quartile3) for continuous variables, and differences between groups were assessed using a t-test or Mann–Whitney U rank-sum test according to distribution status. All of the SNPs were assessed for deviations from Hardy-Weinberg disequilibrium with the use of a χ^2^ test. If the minor SNP genotype frequency was less than 5%, the minor genotype was combined with the intermediate genotype.

Survival curves were generated using the Kaplan–Meier method and compared using the Log-rank test; univariate and multivariate Cox proportional hazard regression was used to find associations with baseline clinical characteristics and 2-year incidence of MACE. Variables with p values < 0.10 were entered into the multivariable model, and variables with p values < 0.10 remained in the model. Because the rs6415084 minor genotype (TT) frequency was less than 5% and only two rs3798220 and rs10455872 genotypes were detected in our subjects, a dominant effect model of Cox regression analysis was used to calculate the hazard ratio (HR) of separate SNP to the incidence of MACE and corresponding 95% confidence interval (CI). The wild-type genotype (W)/W was coded as 0, and the W/variant (V) and V/V genotypes combined were coded as 1. The significance level for all of the statistical tests was P < 0.05. Data analyses were performed using SPSS version 17.0 (Statistical Product and Service Solutions, Chicago, Illinois, USA).

## Results

### *LPA* SNP genotype and allele frequency distribution

The *LPA* SNP genotype and allele frequency distribution are demonstrated in Table 1. Of the three SNPs studied, both rs6415084 and rs3798220 were prevalent in our cohort except rs10455872, for which the variant allele was present in only two patients (G). The variant rs6415014 (T), rs3798220 (C) and rs10455872 (G) allele frequencies were 0.113, 0.081 and 0.002, respectively. There were no significant differences in genotype and allele frequencies between patients with or without MACE (all p > 0.05).

**Table 1:** **Genotype and allele frequency distribution of *LPA *SNPs in CAD patients**

**Subjects**		**All patients**	**Patients without MACE, n = 415**	**Patients with MACE, n = 102**	**P**
		**N = 517**			
**SNP ID**		**n (%)**	**n (%)**	**n (%)**	
rs6415084	Genotype				
	CC	406 (78.5)	367 (78.6)	39 (78.0)	0.250
	CT	105 (20.3)	94 (20.1)	11 (22.0)	
	TT	6 (1.2)	25 (1.3)	0 (0.0)	
	Allele				
	C	917 (88.7)	828 (85.2)	89 (89.0)	0.302
	T	117 (11.3)	144 (14.8)	11 (11.0)	
rs3798220	Genotype				
	CC	433 (83.8)	393 (84.2)	40 (68.1)	0.449
	CG	84 (16.2)	74 (15.8)	10 (21.3)	
	Allele				
	C	950 (91.9)	860 (91.1)	74 (84.1)	0.870
	G	84 (8.1)	74(8.9)	14 (15.9)	
rs10455872	Genotype				
	AA	515 (99.6)	465 (99.6)	50 (100.0)	1.000
	AG	2 (0.4)	2 (0.4)	0 (0.0)	
	Allele				
	A	1032 (99.8)	932 (99.8)	100 (100.0)	1.000
	G	2 (0.2)	2 (0.2)	0 (0.0)	

Abbreviations: *CAD* coronary artery disease, *LPA* Lp(a) gene, *MACE* major adverse cardiovascular event, *SNP* single nucleotide polymorphism.

### The effect of *LPA* SNPs on serum Lp(a) levels in Chinese Han CAD patients

Patients with the CT/TT genotype had a significantly higher Lp(a) levels compared with those that have the rs6415084 CC genotype [41.3 (20.8, 74.6) vs. 18.6 (10.3, 40.9) mg/dl, p < 0.001]. However, there were no significant differences in Lp(a) levels between patients with CC and CG genotype of rs3798220 [23.2 (11.3, 51.9) vs. 18.6 (9.9,46.0) mg/dl, p = 0.411] (Table 2).

**Table 2:** **Serum Lp(a) levels in patients carrying different *LPA *SNP genotypes**

**SNP**	**rs6415084**		**p**	**rs3798220**		**p**
**genotype**	**CC**	**CT/TT**		**CC**	**CG**	
Lp(a) (mg/dl)	18.6	41.3	<0.001	23.2	18.6	0.411
	(10.3, 40.9)	(20.8, 74.6)		(11.3, 51.9)	(9.9,46.0)	

Abbreviations: *LPA* Lp(a) gene, *SNP* single nucleotide polymorphism.

### Baseline characteristics and their effect on MACE

A total of 517 patients made up our study cohort. During a 2-year follow-up, 102 patients (19.7%) suffered at least one incidence of MACE (12.0% coronary revascularization, 3.9% cardiac death, 1.7% non-fatal myocardial infarction, 1.9% ischemic stroke). The baseline characteristics of patients with or without MACE are described in Table 3. Multivariable Cox regression analysis demonstrated that systolic blood pressure (adjusted HR, 1.02; 95% CI: 1.01-1.03), LVEF (adjusted HR, 0.97; 95% CI: 0.96-0.99), use of β-blockers (adjusted HR, 0.62; 95% CI: 0.39-0.97) and elevated Lp(a) levels (≥30 mg/dl) (adjusted HR, 1.77; 95% CI: 1.19-2.63) were independent MACE predictors (Table 4).

**Table 3:** Baseline characteristics in CAD patients with or without MACE

**Characteristics**	**MACE**		**p**
	**Present, n = 102**	**Absent, n = 415**	
Age (yrs)	64.8 ± 12.1	63.1 ± 11.2	0.168
Male (%)	86.3	81.4	0.251
Smokers (%)	30.4	39.3	0.155
Hypertension (%)	66.7.00	56.70	0.105
Diabetes mellitus (%)	30.4	21.9	0.071
History of AMI (%)	35.5	36.8	0.782
History of stroke	4.9	4.6	0.798
Previous PCI	22.5	15.7	0.097
SBP (mmHg)	134.9 ± 23.2	129.7 ± 20.4	0.022
Body mass index (kg/m^2^)	20.8 ± 7.6	21.4 ± 8.2	0.751
Total cholesterol (mmol/L)	4.39 ± 1.27	4.44 ± 1.22	0.681
Triglycerides (mmol/L)	1.17 (0.87, 1.69)	1.30 (0.98, 1.81)	0.306
LDL-C (mmol/L)	2.71 ± 1.04	2.75 ± 0.99	0.729
HDL-C (mmol/L)	1.08 ± 0.29	1.10 ± 0.30	0.627
Lp(a) (mg/dl)	36.3 (12.8, 56.4)	22.4 (10.5, 45.7)	0.012
hs-CRP (mg/l)	3.34 (1.48, 6.80)	3.33 (1.35, 10.80)	0.200
eGFR (ml/min/1.73 m^2^)	92.9 ± 34.1	97.5 ± 33.7	0.228
HbAc1 (%)	6.52 ± 0.98	6.41 ± 1.21	0.496
LVEF (%)	56.0 ± 13.7	59.8 ± 10.9	0.003
Coronary type			
Stable angina	33.3	22.9	0.029
Unstable angina	28.4	39.5	0.038
AMI	38.2	37.6	0.904
Number of lesion vessels	2.34 ± 0.76	2.15 ± 0.81	0.034
Three-vessel disease	52.0	41.9	0.067
Left main disease	16.7	11.8	0.188
Statins	100	98.1	0.366
β-blockers	74.5	81.4	0.103
ACE inhibitors/ARB	81.4	80.7	0.881

Abbreviations: *CAD* coronary artery disease, *MACE* major adverse cardiovascular event, *AMI* acute myocardial infarction, *SBP* systolic blood pressure, *ACE* angiotensin converting enzyme, *ARB* angiotensin receptor blocker, *LDL-c* low density lipoprotein cholesterol, *HDL-c* high density lipoprotein cholesterol, *LVEF* left ventricular ejection fraction, *hs-CRP* high sensitive reactive protein, *eGFR* estimated glomerular filtration rate, *PCI* percutaneous coronary intervention.

**Table 4:** Univariate and multivariate analysis for 2-year MACE incidence

**Univariate analysis**	**HR (95% CI)**	**P**
Diabetes	1.54 (1.01-2.34)	0.047
Previous PCI	1.56 (0.98-2.49)	0.060
Number of vessels with lesions	1.32 (1,03-1.70)	0.030
β-blocker use	0.65 (0.42-1.02)	0.060
SBP	1.01 (1.01-1.02)	0.026
Lp(a) ≥ 30 mg/dL*	1.54 (1.05-2.27)	0.029
LVEF	0.98 (0.96-0.99)	<0.001
Multivariate analysis		
Diabetes	1.50 (0.98-2.30)	0.061
Previous PCI	1.51 (0.95-2.41)	0.085
β-blocker use	0.62 (0.39-0.97)	0.035
SBP	1.02 (1.01-1.03)	<0.001
Lp(a) ≥ 30 mg/dL*	1.77 (1.19-2.63)	0.005
LVEF	0.97 (0.96-0.99)	<0.001

*Lp(a)<30 mg/dl was used for reference, Multivariate analysis was adjusted for diabetes, previous PCI, number of lesioned vessels, β-blocker use, SBP, LVEF and Lp(a) ≥ 30 mg/dL. Abbreviations: *LVEF* left ventricular ejection fraction, *MACE* major adverse cardiovascular event, *SBP* systolic blood pressure, *PCI* percutaneous coronary intervention.

### The effects of *LPA* SNPs on MACE incidence

**Figure 1:**
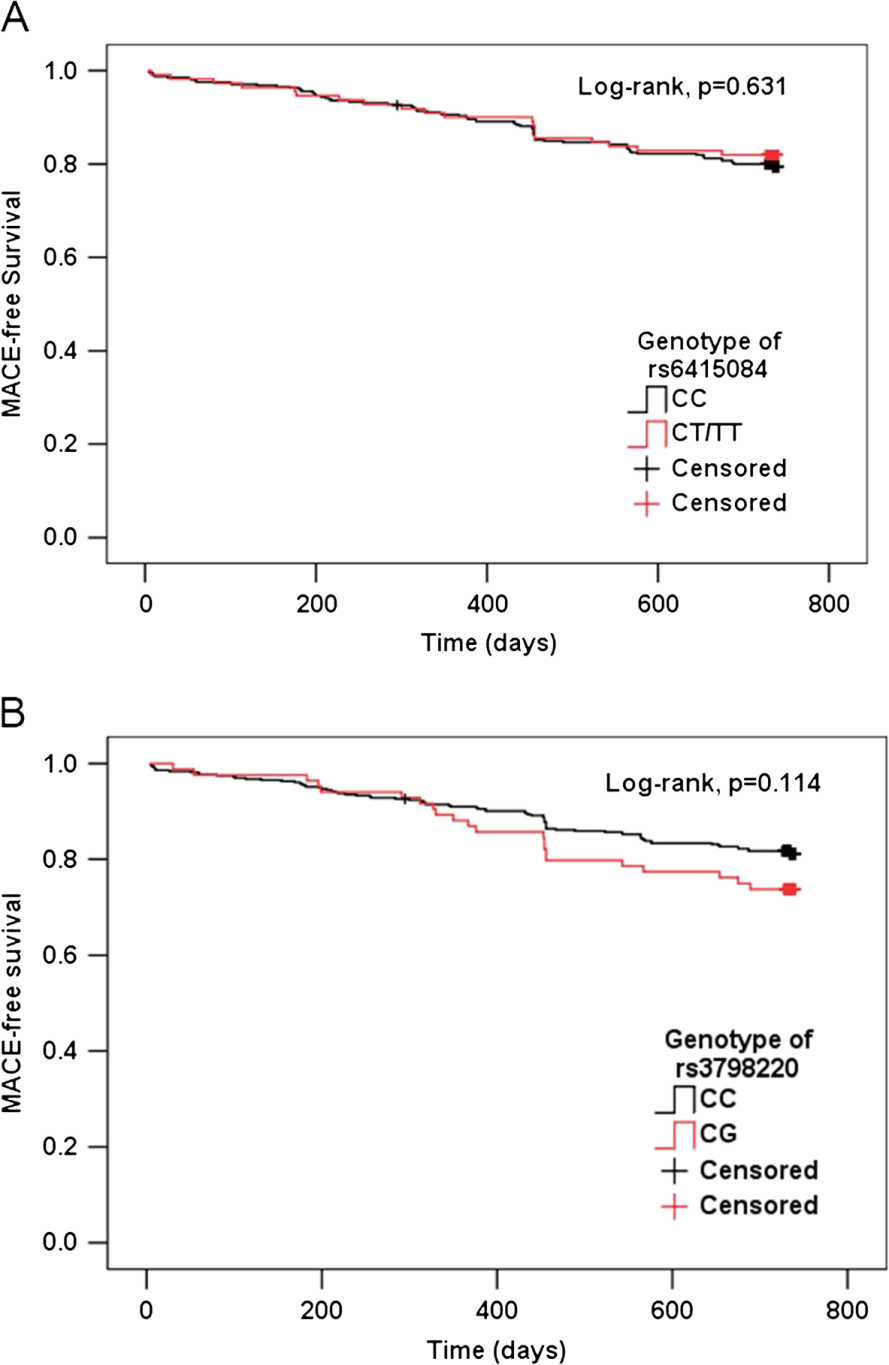
**Kaplan-Meier survival curves of *LPA *SNPs and time to MACE.** Panels **A**, **B** demonstrate the Kaplan-Meier MACE survival curves according to rs6415084 and rs3798220 genotypes, respectively. MACE: major adverse cardiovascular events, including cardiac death, nonfatal myocardial infarction, ischemic stroke and coronary revascularization.

Figure 1 presents the Kaplan–Meier survival curves for the separate SNPs according to their genotypes. There were no significant differences in time to MACE according to the genotypes of separate SNPs (all p > 0.05). Cox regression analysis demonstrated that there were no significant associations between the *LPA* genetic variants (rs6415084 and rs3798220) and time to MACE; the adjusted HR (95% CI) for rs6415084 and rs3798220 were 0.91 (0.55-1.50) and 1.51 (0.92-2.45), respectively (Table 5).

**Table 5:** Association between Lp(a) genetic variants and 2-year MACE incidence

**SNP ID**	**Genotype**	**Frequency**	**MACE**	**HRadj**	**p**
			**n, (%)**	**(95% CI)**	
rs6415084	CC	406 (78.5)	82 (20.2)	0.90	0.681
	CT/ TT	111 (21.5)	20 (18.0)	(0.55-1.48)	
rs3798220	CC	433 (83.8)	80 (18.5)	1.50	0.092
	CG	84 (16.2)	22 (26.2)	(0.94-2.41)	

HRadj: use a multivariate Cox regression model that has been adjusted for age, diabetes, hypertension, previous PCI, 3-vessel disease, β-blocker use, total cholesterol, eGFR, LVEF and coronary artery disease type.

## Discussion

In our study, we genotyped three *LPA* SNPs and evaluated the association of these SNPs with Lp(a) levels in Chinese Han CAD patients. We found that only the rs6415084 variant correlated to Lp(a) levels; we further explored the association of the two SNPs that were most prevalent in our cohort (rs6415084 and rs3798220) and found no association between these SNPs and subsequent cardiovascular event incidence after PCI.

Serum Lp(a) levels are, to a large extent, genetically determined by *LPA* variations in many populations [11,12], and genetic *LPA* variations have been linked directly with CVD risk [5,7]. Notably, certain genetic *LPA* variants, such as SNPs rs3798220 and rs10455872, are strongly associated with both increased Lp(a) levels and CAD risk in Caucasians [17]. However, because of race differences, the genotype frequencies may vary across different races [11,12,17]. As an example, the rs10455872 variant is prevalent and significantly associated with elevated Lp(a) levels in Caucasians; however, the variant allele frequency (G allele) of this SNP is very low (<1%) in Chinese people without CAD [12]. We also found a similar low frequency of this SNP variant allele in our study cohort with CAD. The rs3798220 minor allele (G allele) frequency in the Chinese is relatively higher than the Caucasians (8.1% vs. 1.4%); however, this variation was not associated with elevated Lp(a) levels in Chinese Han CAD patients. The reason for the lack of association is uncertain and may be partially explained by allele frequency differences or apo(a) size heterogeneity, which is the result of a genetically determined functional copy number variation within *LPA*[20], but these hypotheses must be tested in further studies. In our study, rs6415084 was the only SNP that was associated with serum Lp(a) levels, which is consistent with a population-based study [12]. Interestingly, the rs6415084 variant allele frequency in our study was 0.11, which was similar to that of Chinese people without CAD in a SHARE cohort [12,21]. These data suggest that this SNP may not play an important role in CAD pathogenesis, but this hypothesis must be assessed in further studies.

Accumulating evidence has suggested that Lp(a) plays an important role in promoting cardiovascular disease. Elevated Lp(a) levels (≥30 mg/dl) reportedly predict subsequent cardiovascular event incidence in Caucasians with stable coronary disease, especially in patients with suboptimal LDL-C control (≥70 mg/dl) [10]. Recently, Zhou et al. [9] determined that Lp(a) levels at admission are an independent risk factor for subsequent adverse cardiovascular events in Chinese patients with acute coronary syndrome, especially in patients younger than 60 years old. In our study, we found a similar association between elevated Lp(a) levels (≥30 mg/dl) and subsequent adverse cardiovascular events in CAD patients after PCI; elevated Lp(a) levels were associated with 1.77-fold greater likelihood of MACE. Our data and others support the notion that Lp(a) may be used for risk stratification and outcome evaluations.

Although *LPA* SNP variants correlated with elevated Lp(a) levels [12,17], which were associated with high MACE incidence for CAD patients after PCI, there was no direct association between *LPA* SNP variants and MACE incidence. *LPA* variants rs3798220 and rs10455872 have been strongly associated with both increased Lp(a) levels and increased CAD risk [17]. However, because of racial differences, the rs3798220 and rs10455872 variant allele frequency in our cohort was different from the frequency in Caucasians. Both of these SNPs had no significant effect on serum Lp(a) levels in our cohort; therefore, one would not expect that they would have an effect on MACE incidence. Although the rs6415084 variant was strongly associated with higher Lp(a) levels, we did not discover a direct association between rs6415084 polymorphisms and MACE incidence during a 2-year follow-up. Our result is similar to a study by Nicholls et al., who determined that *LPA* SNPs rs2048327, rs3127599, rs7767084 and rs10755578 as well as haplotypes correlated to plasma Lp(a) levels, and Lp(a) levels ≥30 mg/dl were associated with MACE in stable CAD patients during a 3-year follow-up; however, this group did not observe any association of the *LPA* SNPs/haplotypes with increased prospective MACE risk.

CAD is a multi-factorial disease, and multiple genetic variations combined with environmental factors may result in phenotypic variability; therefore, factors that cause cardiovascular events in CAD are complicated. Compared to risk factors such as Lp(a) levels, disease severity, therapeutic reactions and therapeutic adherence are more effective measures for predicting CAD prognosis. In our study, elevated Lp(a) levels were an independent MACE predictor; however, elevated Lp(a) levels were a result of each Lp(a) genetic variant but not any specific SNP. Of the SNPs, rs3798220 explained approximately 8% of total (both genetic and individual-specific) variation in Lp(a) levels, and rs10455872 explained approximately 25% of the variation. Together these two SNPs explained 36% of the variation in Lp(a) levels in Caucasians [17]; however, in our study, rs6415084 explained only approximately 4% of total variation in Lp(a) levels (data not shown). Thus, it was not surprising that there was no significant association between the rs6415084 variant and MACE incidence. To date, our data and others do not support use of Lp(a) genetic variants for CAD risk stratification and outcome evaluations. However, because only three *LPA* SNPs were genotyped, and a relatively small patient sample size was involved in present study, further studies with more *LPA* SNPs and larger patient sample sizes will be required to confirm this conclusion.

## Conclusions

Taken together with previous and present studies, our data did not support a relationship between genetic *LPA* variants rs6415084, rs3798220 and subsequent cardiovascular event incidence after PCI in Chinese Han CAD patients; however, other studies will be required to confirm these findings.

## Competing interests

The authors declared no conflict of interests.

## Authors’ contributions

The study was designed by YZ and ZL. All subjects data were obtained by ZL, GL, ZC, JY, YZ and SS. Experimental data was obtained by ZL and SZ. Data analyses were performed by ZL and SZ. The paper was written by ZL and SZ and all authors read and approved the final manuscript.
